# Predictive Factors for Risk of Reinfection in Septic Two-Stage Revision of Total Hip and Knee Arthroplasties

**DOI:** 10.3390/antibiotics14020167

**Published:** 2025-02-08

**Authors:** Benedikt Paul Blersch, Florian Hubert Sax, Philipp Schuster, Bernd Fink

**Affiliations:** 1 Department of Trauma and Reconstructive Surgery, BG Klinik, University of Tübingen, Schnarrenbergstraße 95, 72076 Tübingen, Germany; bblersch@bgu-tuebingen.de; 2 Department of Joint Replacement, General and Rheumatic Orthopaedics, Orthopaedic Clinic Markgröningen gGmbH, Kurt-Lindemann-Weg 10, 71706 Markgröningen, Germany; florian.sax@rkh-gesundheit.de (F.H.S.); philipp.schuster@rkh-gesundheit.de (P.S.); 3 Department of Orthopaedics and Traumatology, Paracelsus Medical University, Prof. Ernst Nathan Strasse 1, 90419 Nuremberg, Germany; 4 Orthopaedic Department, University Hospital Hamburg-Eppendorf, Martinistrasse 52, 20246 Hamburg, Germany

**Keywords:** septic revision arthroplasty, two-stage revision, predictive factors

## Abstract

Background: The two-stage septic exchange is the most common therapy concept in the treatment of periprosthetic hip and knee infections. However, before the second-stage reimplantation can be carried out, the physician has to assess whether or not the eradication of the periprosthetic joint infection (PJI) has been successful. Therefore, the aim of this study was to evaluate possible predictive parameters for the successful treatment of PJI before and at the time of reimplantation. Methods: This study investigated a total of 145 patients with periprosthetic hip infection and 93 patients with periprosthetic knee infection, who all underwent a two-stage septic exchange between 2017 and 2021. In order to identify possible risk factors for reinfections, the patients underwent preoperative examination of serological inflammatory parameters, microbiological and histological examination of the periprosthetic membrane at the time of reimplantation, as well as postoperative evaluations at regular intervals for a period of at least 24 months. Results: During the follow-up period, reinfection occurred in 11.3% of cases after the two-stage septic revision. None of the serological, microbiological, or histological parameters were able to significantly predict the risk of reinfection. Risk factors associated with reinfection were BMI and previous revision surgery. Conclusions: Currently, there is no reliable predictive factor indicating the risk of reinfection at the time of reimplantation. New diagnostic methods need to be developed to evaluate the possibility and timing of endoprosthesis reimplantation.

## 1. Introduction

Periprosthetic infections are one of the most serious complications following hip and knee prosthesis implantation. A general distinction is made between early infections shortly after implantation and late infections, with the boundary between the two usually being drawn at 4 weeks after implantation [1,2,3]. While in early infections there is a realistic chance of leaving the implant in place and controlling the infection by means of so-called DAIR (debridement, antibiotics, and implant retention), in cases of periprosthetic late infections, the implant and all foreign material must be removed [3]. The so-called two-stage septic exchange is the most common treatment concept in which, after removal of all foreign material, either an antibiotic-releasing spacer is inserted for usually from 6 to 12 weeks or, in the case of the hip, a so-called Girdlestone situation, without a spacer, is created for the same period [3]. If the infection appears to be under control, a new prosthesis is then implanted. Prior to reimplantation, assessing whether an infection has been eradicated or not presents considerable problems for the surgeon. In several studies, our working group and others have shown that the serological inflammation parameters assayed prior to reimplantation have no predictive value [4,5,6,7,8]. Straub et al. [8] were also able to show that the histological processing of the spacer membrane and synovial membrane has no predictive value. It is, therefore, unclear whether there is a predictive factor at all that can give the treating physician an indication as to whether the infection has been controlled—and the new prosthesis can be implanted—or whether debridement and possibly a spacer exchange should be performed again.

The aim of this study was, therefore, to analyze whether any of the usual infection parameters (serological parameters, histological examination of the synovium and the spacer membrane, and bacteriological examination of this tissue) can provide information about the probability of reinfection and act as a predictor for possible reinfection.

## 2. Results

Reinfection occurred in 27 patients (11.3%) during the follow-up period of the two-stage septic revision.

In 17 cases (63.0%), a pathogen shift was observed (Table 1). Identical pathogens were detected in four patients with reinfection (14.8%, Table 2). In four cases (14.8%), a culture-negative infection was initially identified, and, upon reinfection, a pathogen was detected. After an initial pathogen detection, culture-negative reinfection occurred in two cases (7.4%, Table 3).

Of the possible predictive and influencing factors, the following six factors had a *p*-value of <0.30 (Table 4). The possible predictive and influencing factors are shown in Table 5, Table 6 and Table 7. No serological, microbiological, or histological factors were predictive with any level of significance. Only BMI and previous revision had a significant predictive value for reinfection. If revision had been performed before, the risk of reinfection was increased 8-fold. However, the *p*-value was not significant if two of the five microbiological samples were positive, even though the risk of reinfection was increased 4-fold. Similarly, if Krenn type II or III was seen in the histology, the risk was doubled, but there was no significant predictive value.

## 3. Discussion

This study shows that neither the serological parameters nor the bacteriological and histological examination of the synovial and spacer membrane can provide a clear indication of whether there is a risk of reinfection and, therefore, whether reimplantation can be performed at the second stage of the septic two-stage exchange. Thus, these data are unable to identify a factor that can predict whether reimplantation can be performed or not. It was only shown that patients with a previous septic or aseptic exchange operation had an 8-fold increased risk of reinfection after a two-stage septic exchange. Furthermore, patients with a high BMI had a significantly higher risk of reinfection, although this is not helpful in determining the optimal time for reimplantation. Prior revision was also found to be a risk factor for reinfection in the studies by Logroscino et al. [9] and Wang et al. [10] and obesity in the studies by Jhan et al. [11] and Logroscino et al. [9].

In other studies, neither serological parameters [4,5,6,7] nor histological parameters [8] were found to have a significant predictive value. There are several explanations for this. Firstly, a distinction must be made as to whether there is a reinfection with the same pathogen or with a different pathogen. The former can be explained by the presence of osteitis, in which bacteria may be present intracellularly and so would not necessarily be affected during the first stage of treatment. If the immune system is subsequently weakened, this could then lead to an exacerbation of the infection and, thus, reinfection with the same pathogen that caused the first periprosthetic infection [12]. This view is substantiated by the significantly higher risk of reinfection in patients who have already undergone septic replacement surgery. In the present study, 14.8% of patients with reinfection had the same pathogen as in the first septic revision.

Secondly, it is also possible for a new infection caused by other bacteria to develop later, after the successful treatment of the first infection. The patient’s general risk factors and comorbidities for infections play a decisive role here. Reinfection with a different bacterium was seen in 17 cases (63.0%) in our study.

Chen et al. considered intraoperative positive culture as a significant risk factor for reinfection [13]. In our study, the risk of reinfection was increased 4-fold when bacteria were detected in two of the five samples, but this association was not significant in our study.

This study has some strengths and weaknesses. The strength is that a standardized treatment regimen was used in all patients, which has shown success rates of between 92% and 100% in previous studies [3,14,15]. The retrospective evaluation, which does not rule out a certain bias, can be seen as a weakness. A higher number of cases could possibly increase the statistical power due to the higher number of reinfections. However, it seems unlikely that different results can then be expected.

## 4. Materials and Methods

The study design is retrospective: First, the patient data were captured prospectively in the database of the hospital information system. For the current study, the data were retrospectively analyzed. All reviewed patients had received two-stage revision surgery following confirmed PJI of their THA or TKA between 2017 and 2021 in the Orthopaedic Clinic Markgröningen, Germany. Periprosthetic joint infection was diagnosed preoperatively in all cases according to the criteria of the Musculoskeletal Infection Society (MSIS) and the International Consensus on Musculoskeletal Infection (ICM) 2018 [16,17]. Patients with a follow-up of fewer than 24 months and cases with histopathological examination without classification of the periprosthetic membrane, according to Krenn and Morawietz, at the time of reimplantation, were excluded. The medical records of all patients were reviewed for sex, age, body mass index (BMI), time from primary implantation to revision, American Society of Anesthesiologists (ASA) score, Charlson Comorbidity Index (CCI) score, comorbidities, prior revision surgeries (septic, aseptic), type of infection (monobacterial or polybacterial), type of pathogen (easy-to-treat, difficult-to-treat (according to published reports), or methicillin-resistant staphylococci), histopathological examination (neutrophil granulocytes per high-power field, classification of the periprosthetic membrane according to Krenn and Morawietz), microbiological examination, laboratory parameters (white blood cell count, C-reactive protein), and follow-up and outcome (later reinfection or no reinfection).

The groups no reinfection and reinfection were subdivided and then compared according to the predetermined objectives. Statistical analyses were performed as described in Section 4.5.

### 4.1. Characteristics of the Patient Cohort

This study investigated 145 patients (60.9%) with periprosthetic infection in total hip arthroplasty and 93 patients (39.1%) with periprosthetic infection in total knee arthroplasty. The mean age of the 135 male (56.7%) and 103 female (43.3%) patients was 69.4 ± 11.4 (27.8–96.8) years. The body mass index (BMI) of the patient cohort averaged 30.2 ± 6.8 (17.7–60.6) kg/m^2^. Concerning the American Society of Anesthesiologists (ASA) risk classification, 6 patients (2.5%) were classified as ASA I, 102 patients (42.9%) as ASA II, 124 patients (52.1%) as ASA III, and 6 patients (2.5%) as ASA IV [18,19].

Regarding the Charlson Comorbidity Index (CCI), 12 patients (5.0%) were categorized as CCI 0, 15 patients (6.3%) as CCI 1, 41 patients (17.2%) as CCI 2, 47 patients (19.7%) as CCI 3, 48 patients (20.2%) as CCI 4, 36 patients (15.1%) as CCI 5, 24 patients (10.1%) as CCI 6, 8 patients (3.4%) as CCI 7, 3 patients (1.3%) as CCI 8, 2 patients (0.8%) as CCI 9, 1 patient (0.4%) as CCI 10 and 1 patient (0.4%) as CCI 11. In terms of secondary diseases potentially relevant to the development of PJI, 11 patients (4.6%) had a rheumatic disease and 50 patients (21.0%) had diabetes mellitus [19,20].

The average time between the primary implantation and the subsequent two-stage septic revision was 95.9 ± 85.407 (2–500) months. The septic revision was carried out in 70 patients (29.4%), and 13 patients (5.5%) had aseptic revision prior to the analyzed two-stage septic revision.

### 4.2. Characteristics of the Periprosthetic Joint Infections

The periprosthetic joint infections were monobacterial in 201 cases (84.5%) and polybacterial in 37 cases (15.5%). Easy-to-treat (ETT) pathogens were found in 191 infections (80.3%), difficult-to-treat (DTT) pathogens in 21 infections (8.8%), and methicillin-resistant staphylococci (MRS) in 26 infections (10.9%). In 172 cases (72.3%), the histopathological samples obtained during spacer explantation and endoprosthesis reimplantation did not show any evidence of a persisting periprosthetic joint infection: histopathologically, the periprosthetic membrane was classified, in 145 cases (60.9%), as type IV according to Krenn and Morawietz (indifferent type) and, in 27 cases (11.3%), as type I according to Krenn and Morawietz (wear particle type) [21]. A histopathological correlate of a persisting periprosthetic joint infection was identified in 66 cases (27.7%): the periprosthetic membrane showed, in 52 cases (21.8%), type II according to Krenn and Morawietz (infectious type) and, in 14 cases (5.9%), type III according to Krenn and Morawietz (combined type).

Evidence of a persisting periprosthetic joint infection in the microbiological samples from spacer explantation and endoprosthesis reimplantation (identical pathogen detection in at least two out of five microbiological samples) was found in eight patients (3.4%). The average number of neutrophil granulocytes per high-power field detected at the histopathological examination was 6.0 ± 16.2 (0–100).

The preoperative white blood cell (WBC) count averaged 6.4 ± 1.9 (3.1–12.7) 10^3^/µL. The preoperative C-reactive protein (CRP) average was 13.7 ± 15.7 (0–89) mg/L.

### 4.3. Treatment Protocol

The diagnosis of a periprosthetic infection was determined according to the MSIS and ICM criteria with joint aspiration and/or joint biopsy performed prior to any revision endoprosthetic procedure.

A two-stage septic exchange procedure was performed in cases of periprosthetic joint infection. In the stage-one surgery, explantation of the infected endoprothesis, radical debridement, and the implantation of spacer components were performed. Furthermore, samples for microbiological and histological examination were taken intraoperatively. The Krenn and Morowitz type classification [21] was used for the histological examination of the spacer membrane. The spacer consisted of a hip stem coated with an individualized multiantibiotic-loaded bone cement, as previously described [3,14,15]. The bone cement was tailored according to the resistance profile of the pathogen isolated preoperatively (Table 7).

The postoperative antibiotic therapy was also in accordance with the antibiogram and the recommendation of a microbiological consultant. An initial parenteral antibiotic therapy for two weeks was followed by an oral antibiotic therapy for four weeks (Table 8 and Table 9).

The stage-two surgery took place after an interim period of 6 weeks and included explantation of the spacer components, repeated radical debridement, and reimplantation of the endoprothesis. The implant fixation in total hip revision arthroplasty was always performed as a cementless procedure. Cementless fixation was chosen because the typically sclerotic and smooth bone following spacer explantation exhibits impaired cement interdigitation [3,14]. However, solely cementless fixation in total knee arthroplasty revision is not successful at the second-stage surgery, so fixation with cement was chosen for reimplantation of knee prostheses [3]. The cemented fixation was performed with the same mixture of multiantibiotic-loaded bone cement used in the previous stage-one surgery. The antibiotic treatment protocol after the step-two surgery was identical to the preceding antibiotic therapy during the interim phase: two weeks of parenteral antibiotic therapy were followed by oral administration for four weeks.

### 4.4. Laboratory Parameters

The measurement of WBC count (/µL) was performed using a completely automated hematology analyzer (UniCel DxH 800; Beckman Coulter, Pasadena, Calefornia, USA) that utilizes impedance technology and light scatter for cell identification. CRP levels (mg/L) were quantified by the principle of particle-enhanced turbidimetric immunoassay (Cobas C303; Roche, Basel, Switzerland). The recommendations of the manufacturers of UniCel DxH 800 and Cobas C303 were followed exactly.

The measurement of WBC count (/µL) and CRP levels (mg/L) was carried out preoperatively, especially prior to the stage-two revision surgery. The internal threshold value for diagnosing an infection was set at >10 mg/L for the CRP level and at >9000/μL for the WBC count. The Cobas C303 immunoassay does not differentiate CRP levels within the normal range and displays them as <5 mg/L. For the purposes of statistical analysis, the CRP levels in cases with normal values were virtually set at 0 mg/L.

### 4.5. Tests at Second Stage Revision Surgery

During revision surgery, samples for microbiological examination were extracted from five distinct areas close to the prosthesis (periprosthetic tissue and synovium). Additionally, five samples from the synovium and periprosthetic connective tissue membrane associated with the loosened prosthesis were procured for histological evaluation. To prevent the distortion of microbiological results, the administration of perioperative antibiotics took place after the sample collection. The samples for microbiological analysis were placed in sterile sample tubes and were sent to the microbiological institute within a one-hour timeframe. The same procedure was used for synovial fluid samples obtained pre-operatively.

The microbiological samples were inoculated onto blood agar and into nutrient broth specific for anaerobic pathogens. The incubation period was 14 days [22].

The pathogens were categorized into three groups according to Faschingbauer et al. [23]. Group 1 consists of difficult-to-treat (DTT) pathogens, encompassing rifampicin-resistant staphylococcus, quinolone-resistant Gram-negative bacteria, enterococcus, and candida. Methicillin-resistant staphylococci (MRS), including methicillin-resistant staphylococcus aureus (MRSA) and methicillin-resistant staphylococcus epidermidis (MRSE), are classed as group 2. Group 3 consists of easy-to-treat (ETT) pathogens, encompassing any remaining pathogen and culture-negative periprosthetic joint infections.

In histological analysis, the classification system of Morawietz and Krenn et al. was used to categorize the periprosthetic tissue [21,24,25]. The periprosthetic tissue was differentiated into four types: the particle type (I), the infection type (II), the combined type (III), and the indeterminate type (IV). The histopathologist also assessed the number of polymorphonuclear leukocytes per high-power microscope field.

The microbiological assessment of the tissue samples and aspiration fluids was carried out according to the ICM criteria [16,17]: this means that, if the cumulative diagnostic score was equal to or greater than 6, a periprosthetic joint infection (PJI) was diagnosed.

Table 10 summarizes the histological, microbiological, and clinical chemistry results.

### 4.6. Follow-Up Examinations

The follow-up examinations were conducted at regular intervals for a period of at least 24 months. First, the patient data were captured prospectively in the database of the hospital information system. In the second step, the data were retrospectively analyzed. The average follow-up period was 56.2 ± 19.8 (24–112) months.

A successfully treated periprosthetic joint infection was defined according to the criteria of Diaz-Ledezma et al. [26]: The patients were classified as free of reinfection if there was clinical absence of PJI for at least 24 months, microbiological absence of PJI for at least 24 months, no subsequent surgical revision because of reinfection and no PJI-related mortality. We employed the MSIS criteria 2014 and the ICM criteria 2018 for the identification of periprosthetic joint reinfection. The internal threshold of the CRP value for detecting a periprosthetic infection was defined at >10 mg/L [26].

Periprosthetic joint reinfections were treated again by a two-step-septic procedure including local antibiotic therapy (bone cement) and systemic antibiotic therapy which was administered intravenously for two weeks and orally for four weeks. Both occurred in accordance with the antibiogram and the recommendation of the microbiological consultant.

### 4.7. Statistical Analyses

IBM SPSS Statistics for Windows (version 24, IBM Crop., Armonk, NY) was used for statistical analysis. To calculate the cumulative survival, the Kaplan–Meier method was utilized. The survival curve is illustrated by a Kaplan–Meier plot. A multivariable binary logistic regression using the all-enter method was performed, with 15 potential predictors for the primary endpoint of re-infection. The sample size was adequate for the performed multivariate regression analysis [27].

The model was then pared down to include only important predictors, removing variables where *p* > 0.300. Odds ratios (ORs) and 95% CIs (confidence intervals) are presented.

Unless otherwise stated, data are presented as mean ± standard deviation (and range) or number (percentage). The significance level was set at *p* < 0.05.

The research was performed following the guidelines of the Declaration of Helsinki. The study protocol was approved by the Ethics Committee of Landesärztekammer Badenwürttemberg (committee’s reference number F-2023-115).

## 5. Conclusions

In conclusion, this study shows that, currently, there is no clinical chemistry, microbiological, or histological factor that can predict the risk of reinfection and, thus, the optimal time of reimplantation in two-stage septic replacement operations of hip and knee prostheses. A previous replacement operation and a high BMI alone indicate a higher risk of reinfection but have no influence on determining the time of reimplantation. This means that the timing of reimplantation is still determined by the specifications for the various concepts of two-stage septic prosthesis replacement. Future comparative studies will have to show whether it makes sense to carry out longer antibiotic therapies in patients with a higher risk of reinfection.

## Figures and Tables

**Table 1:** Cases of reinfection with a pathogen shift.

	Microorganism One (First Septic Revision)	Microorganism Two (First Septic Revision)	Two or More Samples Positive (of 5) at Time of Re-Implantation	Membrane Type 2 or 3 (Krenn) at Time of Re-Implantation	Microorganism One (Second Septic Revision)	Microorganism Two (Second Septic Revision)
1	*Streptococcus agalactiae* (ETT)		no	yes (type II, 7/HPF)	*Staphylococcus aureus* (ETT)	
2	*Staphylococcus aureus* (ETT)		no	yes (type II, 11/HPF)	*Enterococcus faecalis* (DTT)	*Citrobacter koseri* (ETT)
3	*Streptococcus mitis* (ETT)	*Streptococcus oralis* (ETT)	no	no (type I, 4/HPF)	*Staphylococcus epidermidis* (MRS)	
4	*Staphylococcus epidermidis*, rifampicin-resistant *staphylococcus* (DTT)		no	yes (type III, 6/HPF)	*Enterobacter cloacae* (ETT)	
5	*Pseudomonas aeruginosa* (ETT)		no	yes (type II, 16/HPF)	*Staphylococcus epidermidis* (MRS)	*Candida albicans* (DTT)
6	*Streptococcus gordonii* (ETT)		no	no (type I, 0/HPF)	*Streptococcus mutans* (ETT)	
7	*Streptococcus mitis* (ETT)		no	no (type I, 1/HPF)	*Staphylococcus epidermidis* (MRS)	
8	*Staphylococcus capitis* (ETT)	*Staphylococcus epidermidis* (ETT)	no	no (type I, 2/HPF)	*Staphylococcus lugdunensis* (ETT)	
9	*Staphylococcus aureus* (ETT)		no	yes (type II, 13/HPF)	*Raouletta planticola* (ETT)	
10	*Staphylococcus aureus* (ETT)		no	no (type IV, 0/HPF)	*Staphylococcus epidermidis*, rifampicin-resistant *staphylococcus* (DTT)	
11	*Staphylococcus capitis* (ETT)		no	no (type IV, 0/HPF)	*Corynebacterium urealyticum* (ETT)	*Cutibacterium acnes* (ETT)
12	*Cutibacterium acnes* (ETT)		no	yes (type II, 20/HPF)	*Cutibacterium granulosum* (ETT)	
13	*Staphylococcus capitis* (ETT)	*Cutibacterium acnes* (ETT)	no	yes (type II, 5/HPF)	*Staphylococcus epidermidis*, rifampicin-resistant *staphylococcus* (DTT)	
14	*Staphylococcus epidermidis* (MRS)		no	no (type IV, 0/HPF)	*Cutibacterium acnes* (ETT)	
15	*Cutibacterium acnes* (ETT)		no	yes (type IV, 1/HPF)	*Staphylococcus capitis* (ETT)	
16	*Cutibacterium acnes* (ETT)		no	no (type II, 50/HPF)	*Staphylococcus aureus* (ETT)	
17	*Candida albicans* (DTT)		no	no (type IV, 1/HPF)	*Staphylococcus epidermidis* (MRS)	

**Table 2:** Cases of reinfection with identical pathogens.

	Microorganism One (First Septic Revision)	Microorganism Two (First Septic Revision)	Two or More Samples Positive (of 5) at Time of Re-Implantation	Membrane Type 2 or 3 (Krenn) at Time of Re-Implantation	Microorganism (Second Septic Revision)
1	*Staphylococcus aureus* (ETT)	*Finegoldia magna* (ETT)	No	yes (type III, 5/HPF)	*Staphylococcus aureus* (ETT)
2	*Staphylococcus aureus* (ETT)		No	no (type IV, 1/HPF)	*Staphylococcus aureus* (ETT)
3	*Staphylococcus aureus* (ETT)		Yes	no (type IV, 1/HPF)	*Staphylococcus aureus* (ETT)
4	*Eschericia coli*, quinolone-resistant Gram-negative bacteria (DTT)		No	no (type IV, 0/HPF)	*Eschericia coli*, quinolone-resistant Gram-negative bacteria (DTT)

**Table 3:** Cases of reinfection with culture-negative infection in first or second septic revision.

	Microorganism One (First Septic Revision)	Two or More Samples Positive (of 5) at Time of Re-Implantation	Membrane Type 2 or 3 (Krenn) at Time of Re-Implantation	Microorganism One (Second Septic Revision)
1	No pathogen detectable	yes	no (type IV, 1/HPF)	*Pseudomonas aeruginosa* (ETT)
2	No pathogen detectable	no	yes (type II, 25-30/HPF)	*Staphylococcus aureus* (ETT)
3	No pathogen detectable	no	no (type IV, 0/HPF)	*Staphylococcus aureus* (MRS)
4	No pathogen detectable	no	no (type IV, 0/HPF)	*Streptococcus dysgalacticae* (ETT)
5	*Cutibacterium acnes* (ETT)	no	yes (type II, >100/HPF)	No pathogen detectable
6	*Enterococcus faecalis* (DTT)	no	no (type IV, 0/HPF)	No pathogen detectable

**Table 4:** Predictors of reinfection after paring down the model using *p* < 0.300 *.

Predictor Variable	Odds Ratio	95%-CI		*p*-Value
Sex	2.30	0.796	6.625	0.124
BMI	1.01	1.013	1.163	0.020
ASA	1.85	0.654	5.248	0.246
Any revision before	8.05	2.898	22.336	0.001
Krenn Type II or III at time of revision	2.52	0.780	8.116	0.122
≥2 of 5 microbiological samples positive at time of re-implantation	4.21	0.447	39.750	0.209

* Sex included as male vs. female; effect of BMI per increase of one; effect of ASA increase of one category; other factors included as dichotomous variables (yes/no). BMI: body mass index.

**Table 5:** Characteristics of patient cohort.

Cases	238
Sex	
Male	135 (56.7%)
Female	103 (43.3%)
Age [years]	69.4 ± 11.4 (27.8–96.8)
BMI (kg/m^2^)	30.2 ± 6.8 (17.7–60.6)
Time from primary implantation to revision [months]	95.9 ± 85.407 (2–500)
Joint	
Knee	93 (39.1%)
Hip	145 (60.9%)
ASA	
I	6 (2.5%)
II	102 (42.9%)
III	124 (52.1%)
IV	6 (2.5%)
CCI	
0	12 (5.0%)
1	15 (6.3%)
2	41 (17.2%)
3	47 (19.7%)
4	48 (20.2%)
5	36 (15.1%)
6	24 (10.1%)
7	8 (3.4%)
8	3 (1.3%)
9	2 (0.8%)
10	1 (0.4%)
11	1 (0.4%)
Rheumatic disease	
Yes	11 (4.6%)
No	227 (95.4%)
Diabetes	
Yes	50 (21.0%)
No	188 (79.0%)
Septic revision before	
yes, 1 or more septic revisions	70 (29.4%)
No	168 (70.6%)
Aseptic revision before	
yes, 1 or more septic revisions	13 (5.5%)
No	225 (94.5%)

**Table 6:** Characteristics of periprosthetic joint infections.

Type of Infection	
Monobacterial	201 (84.5%)
Polybacterial	37 (15.5%)
Bacteria	
ETT	191 (80.3%)
DTT	21 (8.8%)
MRS	26 (10.9%)

**Table 7:** Type of bone cement.

Copal G+C ^1^	35 (14.7%)
Copal G+V ^2^	9 (3.8%)
Copal G+C, mixed ^3^	186 (78.2%)
Copal G+V, mixed ^4^	7 (2.9%
Palacos +G, mixed ^5^	1 (0.4%)

^1^ rapid-curing, radiopaque bone cement containing gentamicin and clindamycin; 42.7 g of COPAL^®^ G+C powder contains 1.0 g gentamicin (as gentamicin sulfate) and 1.0 g clindamycin (as clindamycin hydrochloride); produced by Heraeus Medical GmbH, Wehrheim, Germany. ^2^ rapid-curing, radiopaque bone cement containing Gentamicin and Vancomycin; 43.0 g of COPAL^®^ G+V cement powder contains: 0.5 g gentamicin (as gentamicin sulfate) and 2.0 g vancomycin (as vancomycin hydrochloride); produced by Heraeus Medical GmbH, Wehrheim, Germany. ^3^ Copal G+C^®^ mixed with (1) vancomycin/(2) meropenem/(3) streptomycin/(4) ofloxacin/(5) fosfomycin/(6) streptomycin/(7) vancomycin, ampicillin/(8) vancomycin, amphotericin B/(9) vancomycin, ofloxacin/(10) clindamycin, gentamicin. ^4^ Copal G+V^®^ mixed with (1) vancomycin/(2) meropenem/(3) cefazolin. ^5^ Palacos +G^®^ (fast-curing, radiopaque bone cement containing 2% gentamicin; produced by Heraeus Medical GmbH, Wehrheim, Germany) mixed with (1) vancomycin/(2) vancomycin, ofloxacin.

**Table 8:** Intravenously administered antibiotics or antimycotics and number.

Antibiotic One	Antibiotic Two	Antibiotic Three	Antibiotic Four	Number
Vancomycin	Rifampicin			54
Flucloxacillin	Rifampicin			43
Ampicillin/sulbactam	Rifampicin			41
Vancomycin	Imipenem			20
Imipenem	Rifampicin			6
Penicillin G	Rifampicin			4
Cefuroxim	Rifampicin			8
Vancomycin	Fosfomycin			8
Levofloxacin	Rifampicin			7
Imipenem/cilastatin	Rifampicin			1
Daptomycin	Rifampicin			5
Vancomycin	Meropenem			4
Ciprofloxacin	Meropenem			3
Vancomycin	Imipenem	Rifampicin		1
Vancomycin	Meropenem	Rifampicin		3
Amoxicillin/clavulansäure	Rifampicin			2
Cefuroxim				1
Flucloxacillin	Piperacillin/tazobactam			2
Meropenem				2
Amoxicillin/clavulansäure	Daptomycin			1
Ampicillin	Rifampicin			1
Ampicillin/sulbactam	Fosfomycin			1
Ampicillin/sulbactam	Imipenem			1
Vancomycin	Ampicillin/sulbactam			1
Ampicillin/sulbactam	Metronidazol			1
Ampicillin/sulbactam	Amikacin	Ethambutol	Pyrazinamid	1
Ampicillin/sulbactam	Clindamycin			1
Cephazolin	Clindamycin			1
Vancomycin	Ciprofloxacin			1
Cotrimoxazol	Rifampicin			1
Flucloxacillin	Fosfomycin			1
Flucloxacillin	Levofloxacin	Rifampicin		1
Flucloxacillin	Meropenem	Rifampicin		1
Flucloxacillin	Moxifloxacin			1
Vancomycin	Fosfomycin	Imipenem		1
Fosfomycin	Meropenem			1
Gentamicin	Penicillin G			1
Levofloxacin	Meropenem			1
Vancomycin	Levofloxacin			1
Levofloxacin	Metronidazol			1
Penicillin G				1
Voriconazol	Rifampicin			1

**Table 9:** Administered oral antibiotics or antimycotics and number.

Antibiotic One	Antibiotic Two	Antibiotic Three	Antibiotic Four	Number
Levofloxacin	Rifampicin			141
Amoxicillin/clavulansäure	Rifampicin			31
Cotrimoxazol	Rifampicin			11
Linezolid	Rifampicin			6
Amoxicillin/clavulansäure	Levofloxacin			11
Clindamycin	Rifampicin			7
Ciprofloxacin				4
Clindamycin	Levofloxacin			4
Moxifloxacin	Rifampicin			3
Amoxicillin/clavulansäure	Levofloxacin	Rifampicin		1
Ampicillin/Sulbactam	Rifampicin			2
Ciprofloxacin	Linezolid			2
Clarythromycin	Levofloxacin	Rifampicin		2
Cotrimoxazol				2
Levofloxacin				2
Amikacin	Ethambutol	Pyrazinamid	Rifabutin	1
Amoxicillin/clavulansäure	Metronidazol			1
Cefuroxim	Clindamycin			1
Ciprofloxacin	Levofloxacin			1
Cotrimoxazol	Fluconazol			1
Cotrimoxazol	Levofloxacin			1
Cotrimoxazol	Levofloxacin	Rifampicin		1
Levofloxacin	Metronidazol			1
Rifampicin	Voriconazol			1

**Table 10:** Histological examination, microbiological examination, and clinical chemistry.

Membrane Type (Krenn)	
type 1	27 (11.3%)
type 2	52 (21.8%)
type 3	14 (5.9%)
type 4	145 (60.9%)
Neutrophil granulocytes per high-power field	6.0 ± 16.2 (0–100)
Membrane type 2 or 3 (Krenn) at time of re-implantation	
Yes	66 (27.7%)
No	172 (72.3%)
Two or more samples positive (of 5) at time of re-implantation	
Yes	8 (3.4%)
No	230 (96.6%)
WBC at time of re-implantation	6.4 ± 1.9 (3.1–12.7)
CRP at time of re-implantation [mg/L]	13.7 ± 15.7 (0–89)

## Data Availability

We do not wish to share our data because some of the patient’s data relate to individual privacy and, according to the policy of our hospital, these data may not be shared with others without permission.
